# The Personal and Interpersonal Components of Perfectionism: The Italian Validation of “Multidimensional Inventory of Perfectionism in Sport”

**DOI:** 10.3390/ijerph18052657

**Published:** 2021-03-06

**Authors:** Alessandra De Maria, Luca Mallia, Caterina Lombardo, Mariacarolina Vacca, Arnaldo Zelli

**Affiliations:** 1 Department of Movement, Human, and Health Sciences, University of Rome “Foro Italico”, 00135 Rome, Italy; luca.mallia@uniroma4.it (L.M.); arnaldo.zelli@uniroma4.it (A.Z.); 2 Department of Psychology, Sapienza University of Rome, 00185 Rome, Italy; caterina.lombardo@uniroma1.it (C.L.); mariacarolina.vacca@uniroma1.it (M.V.)

**Keywords:** MIPS, perfectionism, sport competitive anxiety, exploratory structural equation modeling

## Abstract

The present research focused on the general theme of perfectionism in the sport domain, and it provided the first empirical validation of the original 72-item “Multidimensional Inventory of Perfectionism in Sport” (MIPS) among Italian athletes. The study, specifically, also focused on the relations linking personal and interpersonal components of perfectionism to athletes’ competitive anxiety. The research overall relied on data from 644 Italian sport science students and professional athletes and included both cross-sectional and longitudinal designs. Data analyses primarily focused on structural equation modeling, and the findings overall supported the psychometric and construct validity of the Italian version of the MIPS, also highlighting the key role of the personal components of perfectionism.

## 1. Introduction

Scientific attention to the construct of perfectionism has a long history, especially in the field of personality research, and perfectionism tends to be viewed as a relatively stable characteristic that can influence behavior in several areas of daily life and experiences, such as, for instance, sport [1].

Despite these well-established general views, scientific literature on perfectionism has been and still is debating some basic issues about the construct, ranging from whether it represents a personality trait or a disposition to which life experiences contribute to its development [2,3].

One issue that scholars have animatedly debated over the decades has to do with the dimensionality of perfectionism, and this issue has been of interest to clinicians as well as personality and developmental psychologists. Illustratively, the early information about perfectionism was based on theorists and clinicians’ impressions [4,5], who considered perfectionism as a one-dimensional construct which may serve self-esteem needs [4], help to overcome self-belittlement [6] or, from a different perspective, reflect parenting styles’ effects into one’s adulthood [5]. The notion of one-dimensional perfectionism has occasionally been resisted until recent times [7] even though, as a whole, most of the current perfectionism research seems to have firmly converged on the alternative notion that perfectionism is a multidimensional construct.

In a very recent contribution, perfectionism is conceptualized and defined as “a multidimensional personality disposition characterized by a striving for flawlessness and setting exceedingly high standards of performance accompanied by overly critical evaluations of one’s behavior” [8] (p. 3). This definition captures the core idea embedded in early efforts [9] posing that one’s tendency to perfectionism may influence personal and interpersonal experiences. Along these lines, researchers tend to agree that there exist two relatively distinct general dimensions of perfectionism, namely, perfectionistic strivings and perfectionistic concerns [10]. Perfectionistic strivings reflect aspects of perfectionism associated with an individual’s tendency to pursue perfection and to set exceedingly high standards of performance. In contrast, perfectionistic concerns reflect aspects of perfectionism associated with one’s concerns over making mistakes, with fears of being negatively evaluated by others, combined with a tendency toward self-criticism [11].

In general, these two general dimensions of perfectionism have shown different and contrasting patterns of relations with various processes and outcomes, despite the fact that they are often highly correlated [12]. Perfectionistic strivings tend to show positive relations with processes or outcomes that typically are valued and considered positively, such as self-determined motivation, optimism, group cohesion, competitive self-confidence and performance [13,14,15,16,17]. In contrast, perfectionistic concerns tend to show positive relations with non-adaptive processes and outcomes, such as motivation that is anchored to environmental influences, competitive anxiety, burnout or sports injury [16,17,18,19].

### 1.1. Multidimensionality of Perfectionism in Sport

These considerations notwithstanding, the notion of the multidimensionality of perfectionism may acquire different meanings in different life and behavioral contexts. That is, the number and types of dimensions characterizing perfectionism may vary across contexts. Dunn and colleagues’ research has overall shown that athletes tend to have higher perfectionism levels in sports than in other areas of life [20]. Moreover, athletes have been found to be higher in perfectionism than the general population [21] and they are more prone to experiencing perfectionistic concerns and perfectionistic strivings-related aspects [22]. Thus, the complexity and salience of the personal and interpersonal experiences that tend to characterize athletes’ lives have solicited sport research scholars to embed the study of perfectionism within a broader spectrum of dimensions [23].

Overall, athletes face multiple situations in their daily experiences, and an athlete’s own tendency to perfectionism strivings or concerns may be experienced quite differently depending upon situational or interpersonal contingencies such as, for instance, training sessions, high-level competitions, teammates or coaches, and parental pressure. The “Multidimensional Inventory of Perfectionism in Sport” (MIPS) [24] is a 72-item instrument that reflects these notions and that envisions perfectionism as emerging from a broad array of personal, situational and interpersonal dimensions characterizing the sport field. 

As to “personal” dimensions, the MIPS considers several dimensions or sub-scales that, overall, tap onto athletes’ adaptive or maladaptive forms of perfectionism during both training sessions and actual competitions, thus articulating the notions of perfectionistic strivings and concerns. In line with this approach, perfectionistic aspirations (PA; also known as striving for perfection in [17]) are key indicators of perfectionistic strivings, whereas negative reactions to non-perfect performance (NR; also named negative reaction to imperfection in [17]) are key indicators of perfectionistic concerns [25].

As to “interpersonal” dimensions, that is, the sub-scales of “perceived pressure from coach” (PPC), “perceived pressure from teammates” (PPT) and “perceived pressure from parents” (PPP), the MIPS has been designed to address the possibility that coaches, teammates or parents represent salient counterparts exerting social pressure on athletes to adopt and seek perfectionism in their sport activities. Parents’ role is considered more relevant for young athletes as they represent their main source of inspiration in early years, followed by coaches and teammates in later adult years [24]. In line with Hewitt and Flett [26], the MIPS also envisions the possibility that athletes’ perfectionism may extend to a sort of social comparison process by which athletes may expect and react to the extent to which significant others (e.g., teammates) embrace and adopt behaviors consistent with perfectionism. This attention led to the formulations of the two MIPS sub-scales of “perfectionistic pressure on teammates” (PPOT) and “negative reactions to non-perfect performance of teammates” (NRTT).

The use of the 72-item MIPS in sport research is not common. MIPS has been initially validated among German athletes by its authors [24] and, to the best of our knowledge, it only has been fully translated in English language to be tested among high school and university athletes [27]. Interestingly enough, there exists a larger body of research on shorter versions of the instrument, in which items either primarily refer to the two broad dimensions of perfectionism, namely, perfectionistic strivings and perfectionistic concerns, or also refer to a sort of dimension concerning “social pressure to be perfect”, in which athletes express their perceptions of their parents and coaches’ solicitations toward perfectionism [25]. These shorter versions of MIPS thus may presuppose four underlying dimensions, that is, two “personal” dimensions of perfectionism (strivings and concerns) and two “interpersonal” dimensions, referring to athletes’ perceived pressure by parents and coaches, respectively. These shorter versions have been translated in several languages and have received ample psychometric and construct (external and predictive) validity evidence, mainly in studies involving adolescent and adult athletes and adopting both traditional and more rigorous statistical approaches to data analyses [25,28,29,30,31].

In the sport domain, the use of shorter versions of MIPS is predominant, and researchers [25] have alerted against the use of other instruments, such as the “Perfectionism in Sport Scale” (PSS) [23], as their sporadic use inevitably leads to a lack of solid evidence on the instruments’ external and predictive validity.

### 1.2. Perfectionism and Anxiety in Competitive Sport

Athletes who play sport at competitive levels often view themselves as perfectionists and consider perfectionism as either a significant source of their success or a pervasive source of personal and interpersonal difficulties [32]. Participating in sport is indeed characterized by a demand to perform to optimum levels in intense pressure situations, where success is required while failure is unwanted. For these reasons, competitive sport has long been better defined as “more than just a game” [33].

The way athletes experience it, especially during competitions and followed by close scrutiny of their coaches, teammates and parents, can be associated with higher levels of competitive anxiety [34]. According to Martens and colleagues [34] competitive anxiety comes from the union of three dimensions: cognitive anxiety, somatic anxiety and self-confidence. The first two dimensions represent, respectively, the mental and physiological aspects of anxiety, whereas self-confidence refers to positive assessments about one’s own capabilities.

To date, several studies have revealed significant relations between athletes’ perfectionism and competitive anxiety. For instance, Hall and colleagues [35] suggested that concerns over personal mistakes (i.e., a component of perfectionistic concerns) among high school runners can act as predictors of cognitive anxiety before performance, whereas personal standards (i.e., a component of perfectionistic strivings) appear to be predictors of athletes’ self-confidence. Likewise, Koivula and colleagues’ study [36] showed that high personal strivings and little concerns over mistakes among Swedish elite athletes (i.e., athletes classified as “positive perfectionists”) predicted relatively high self-confidence and low levels of cognitive and somatic anxiety, as compared to those athletes who exhibited an opposite perfectionism profile (i.e., athletes classified as “negative perfectionists”). This two-fold pattern of relations highlighting the link between strivings for perfection and self-confidence on one side and concerns over “imperfection” and competitive anxiety on the other has been confirmed in other studies conducted with elite athletes [31], although there are empirical exceptions to this general finding ([17]; see also the meta-analytical review by [12]).

### 1.3. The Present Study

The above considerations highlight two broad issues. The first one is concerned with how perfectionism is conceptualized and measured. As we have reviewed, perfectionism may have a multidimensional basis, and both personal and interpersonal experiences with significant others may act as contributing factors. Furthermore, perfectionism tends to coalesce on the two main components of high “perfectionistic strivings” or standards and impairing “perfectionistic concerns”, and it is domain specific, that is, varies across life domains. In this sense, a person might experience and assign to himself or herself perfectionistic tendencies in one domain (e.g., sport) but not in another, as other scholars have also clearly recognized [1,20].

The second related issue is concerned with the relations between perfectionism and anxiety, especially in the context of competitive sport. Researchers have suggested to differentiate between general measures of general perfectionism and sport-specific measures of perfectionism [17,25]. Nevertheless, studies that adopted a sport-specific measure of perfectionism did not provide consistent results, especially with regards to the relations linking perfectionistic strivings and competitive anxiety. The issue of the linkages between perfectionism and competitive anxiety appears even more complex when one considers the limited body of work that has addressed these linkages in the context of the interpersonal sources of influence on athletes’ perfectionism, such as parents, coaches and teammates [37].

Together, the two issues above seem to warrant a close and thorough inspection of the personal and interpersonal components of perfectionism, as outlined in the original version of the MIPS (i.e., nine dimensions, 72 items). This position seems warranted when one also considers that the multidimensional characteristics of perfectionism may help in exploring more thoroughly the relations between perfectionism and competitive anxiety. The present study pursues this general goal, and it adopts the original 72-item version of the MIPS as a conceptually solid instrument to seek empirical evidence on the relations linking athletes’ perfectionism and competitive anxiety.

In doing so, the present study also pursued three distinct ancillary goals. The first one was to assess the psychometric and construct validity of MIPS in an Italian sample of athletes, a first-time empirical endeavor. The second goal was to assess the relations between perfectionism components and competitive anxiety within a longitudinal design, thus overcoming the weaknesses of cross-sectional designs (see [12] for a discussion). The third and related goal was to test the psychometric and construct validity tenets of the MIPS via the use of exploratory structural equation modeling (ESEM) [38], which offers rigorous criteria to estimate measurement error in the input data and evaluate the quality of hypothesized relations among key variables.

## 2. Materials and Methods

### 2.1. Participants

A convenience sample of 644 Italian participants (43.2% females) between the ages of 16 and 35 years (M = 21.7, SD = 3.6) took part in the research. Participants were either sport science students at the University of Rome “Foro Italico” or professional athletes from different regions of Italy. As to the distribution of participants across sports, 46.4% of them practiced individual sports (e.g., swimming, artistic gymnastics, tennis, athletics, etc.), 50.9% practiced team sports (e.g., football, basketball, volleyball, water polo, etc.) and 2.3% practiced both individual and team sports. As to the level of competitive sports, participants practiced at either amateur (27%), local (7.9%), regional (30.6%), national (26.1%) or international (8.4%) levels of competition. Finally, participants have been practicing their sports for an average of 10.5 years (SD = 5.1), and they on average trained 3.6 times a week (SD = 1.6) and for a total of about 8.1 h per week (SD = 5.9). This sample was relatively large to have sufficient data on the instrument (MIPS) which was the key focus of the current study and was considered enough following the general rule of thumb for structural equation modeling (SEM) research, i.e., to include a parameters ratio of 10:1 or at least 5:1 [39].

A subsample of 329 participants (46.2% females), with mean age of 21.5 (SD = 3.9) also provided additional data for the purpose of testing psychometric and validity characteristics of MIPS data. Among this subsample, 46.2% of participants practiced individual sports (e.g., boxing, tennis, cross country skiing, fencing, etc.), 51.7% team sports (e.g., football, basketball, volleyball, rugby, etc.) and 1.8% practiced both individual and team sports, and these sports were practiced at an amateur (26.1%), local (9.7%), regional (28.6%), national (23.7%) or international (11.9%) level of competition. Participants have been practicing their sports for an average of 10.8 years (SD = 5.2), and they on average trained 3.6 times a week (SD = 1.5) and for a total of about 8.2 h per week (SD = 6.6).

### 2.2. Procedures

The present study was approved by the University’s Institutional Review Board (IRB protocol, nr. CAR 09/2019) of the University of Rome “Foro Italico”. Professional athletes voluntarily participated following an e-mail contact with their coaches or sports club managers, whereas university students received an extra point on the final grade for their voluntary participation. All participants, and parents of minors if present, were informed about the purposes of the research and assured about the anonymity and confidentiality of their responses, as well as about the possibility to withdraw from the study at any time. After signing informed consent forms, all participants filled out the questionnaires on an internet webpage (SurveyMonkey) to which they could access via a link sent to their personal e-mails. For the purposes of the current study, the subsample provided additional data on some of the measures in two separate occasions over the course of nearly two months (see following section for details). For this latter subsample, a self-generated code was used to individually match data on the questionnaires collected during the two distinct assessments.

### 2.3. Measures

#### 2.3.1. Descriptive Information

All participants filled out a questionnaire created ad hoc for the present study, providing information on sociodemographic variables, such as gender, age, residence, employment status, and sport-specific information (main sport, years of training, duration of training sessions, number of sessions per week and competitive level).

#### 2.3.2. Sport Perfectionism (MIPS)

All participants provided data at least once on the original Multidimensional Inventory of Perfectionism in Sport (MIPS) [27], which measures the dimensions of perfectionism in sport domains. The MIPS consists of 72 items, divided into nine eight-item subscales assessing “perfectionistic aspirations during training” (e.g., “During training, I feel the need to be perfect”), “perfectionistic aspirations during competitions” (e.g., “During competitions, I feel the need to be perfect”), “negative reactions to non-perfect performance during training” (e.g., “During training, I feel extremely stressed if everything doesn’t go perfectly”), “negative reactions to non-perfect performance during competitions” (e.g., “During competitions, I feel extremely stressed if everything doesn’t go perfectly”), “perceived pressure from coaches” (e.g., “My coach expects my performance to be perfect”), “perceived pressure from teammates” (e.g., “My teammates expect my performance to be perfect”), “perceived pressure from parents” (e.g., “My parents expect my performance to be perfect”), “perfectionistic pressure on teammates” (e.g., ”I demand nothing less than perfection of my teammate”) and “negative reactions to non-perfect performance of teammates” (e.g., “I am dissatisfied with my teammates, even when I know that they are doing their best”). All participants rated all items on a 6-point Likert scale ranging from 1 (rarely) to 6 (always). Evidence for the validity and reliability of the MIPS has been provided by Stoeber and colleagues [27], who have reported Cronbach’s alpha coefficients ranging from 0.88 to 0.95. The subsample provided MIPS data a second time about two months after the first MIPS assessment.

#### 2.3.3. General Perfectionism (F-MPS)

During the first assessment, the subsample participants (n = 329) also provided data on a general measure of perfectionism validated by Lombardo [40] (“Multidimensional Perfectionism Scale”, F-MPS) [9]. It is a 35-item scale capturing “personal standards” (7 items; e.g., “I set higher goals than most people”), “concerns over mistakes and doubts” (14 items; e.g., “If I fail partly, it is as bad as being a complete failure”), “parental expectations and criticism” (8 items; e.g., “My parents have always had higher expectations for my future than I have”) and “organization” (6 items; e.g., “Organization is very important to me”). Personal standards (PS) reflect perfectionists’ exceedingly high standards of performance, whereas concerns over mistakes and doubts (CMD) reflects perfectionists’ fear about making mistakes along with a tendency towards indecisiveness related to an uncertainty about doing the right thing. Parental expectations and criticism (PEPC) reflect perfectionists’ perception that parents set very high goals and are overly critical, and organization (O) reflects tendencies to be organized. Participants rated all items on a 5-point Likert agreement scale ranging from 1 (strongly disagree) to 5 (strongly agree). Lombardo [40] has offered evidence for the validity and reliability of the MPS in an Italian sample (Cronbach’s alpha coefficients ranging from 0.76 to 0.87). In the present study, the F-MPS data provided a means for assessing construct validity of the translated MIPS scale. The F-MPS subscales showed good or very good internal consistency coefficients, as measured by Cronbach Alpha (from 0.74 to 0.88).

#### 2.3.4. Competitive State Anxiety

During the second assessment, the subsample participants (n = 329) provided data on the Italian validated version [41] of the “Competitive State Anxiety Inventory-2” (CSAI-2), originally developed by Martens and colleagues [34]. This instrument was used to assess participants’ state anxiety with respect to sport performance during competitions. The scale comprises 27 items divided in three nine-item subscales assessing, respectively, cognitive anxiety (e.g., “I am concerned about losing”), somatic anxiety (e.g., “I feel tense in my stomach”) and self-confidence (e.g., “I’m confident because I mentally picture myself reaching my goal”). Participants rated each item on a 4-point Likert scale ranging from 1 (not at all) to 4 (very much so), with subscales’ theoretical scores ranging from 9 to 36. Item 14, a reverse-scored item belonging to the “somatic anxiety” subscale, was rescored before analysis. As the inventory was not administered prior to competition, the general statement “How you feel right now” was modified to “Think about your last competition”. The Italian version of the instrument has been considered a reliable and valid measure (documented Cronbach’s alpha 0.85–0.90) of competitive state anxiety. This instrument was employed for the predictive validity assessment of the translated MIPS instrument. In the present study, participants’ data on the three dimensions of cognitive anxiety, somatic anxiety and self-confidence yielded internal reliability coefficients of 0.89, 0.90 and 0.91, respectively.

### 2.4. Cross-National Translation of the MIPS

To rely on a valid and well-adapted version of the original MIPS [27] the authors followed the recommendations of the International Test Commission [42] and adopted a multiple-step approach for the Italian translation of the MIPS. This approach included the back-translation method [43], a review by a committee of experts [44] and a pre-test in a small group. As the first step, two bilingual experts in the field translated the English MIPS and its instructions into Italian language. Afterward, two bilingual academic experts blindly back translated it to the source language. As the second step, the committee of experts, which also included the two translators called upon in the first phase, first assessed the extent to which there were similarities, language equivalency and functionality between the two versions, English and Italian, of the MIPS and then developed upon consensual agreement among the committee experts a first final version of the Italian MIPS. As the third step, this final version was administered to a small group of students to examine the extent to which they understood all items and understanding of all items and to assess the efficacy of the web system used for data collection. At the end of this procedure, experts collegially agreed upon the version of the Italian MIPS that was used in this study.

### 2.5. Data Analysis

The online procedure of data collection was effective and there were no missing data. Prior to data analyses, the distribution characteristics of MIPS item data collected from the entire sample were visually examined, and most of the items had skewness values that were at either or just above the value of 1.

A first series of analyses then examined the factor structure of the MIPS data collected from the entire sample (n = 644). The overall approach followed the procedures of structural equation modeling (SEM). Firstly, the MIPS factor structure was examined with “exploratory structural equation modeling” (ESEM) [38] using Mplus (version 7.0, Muthén & Muthén, Los Angeles, CA, USA) [45]. ESEM is a global exploratory SEM framework that incorporates the best features of confirmatory factor analysis (CFA) and those of exploratory factor analysis (EFA) [38,46], making the advances traditionally associated with CFA available in EFA. Like EFA, ESEM permits the examination of the underlying structure of a scale with loading matrix rotation to obtain an interpretable solution [46]. Additionally, ESEM allows free cross-loadings of any item on multiple factors that are traditionally constrained to be zero in CFA [38]. Evidence has shown that ESEM solutions provide a better fit in relation to CFA solutions and that the overly restrictive assumption of CFA often leads to biased estimates in SEMs [38,46]. However, like CFA, ESEM gives access to all data fit information, such as standard errors and residual correlations, and it also allows the introduction of factors that reflect the method used and not the trait investigated [38]. For these reasons, the flexibility of the model makes ESEM a more suitable statistical technique to study the factor structure of a measure when, as in this case, the dimensionality is still unexplored [38].

ESEM analyses sequentially tested three exploratory models with, respectively, a seven, eight and nine factor solution by means of a maximum likelihood estimator (MLR), estimates of standard errors and tests of fit that are robust for non-normality, and Geomin rotations (the default in the Mplus [38]). This choice was made to compare the nine-factor solution (i.e., what was expected) with solutions that would evaluate the possibility that the Italian sample’s MIPS item data would generate a different number of factors. Conventional fit indices provided a rigorous comparison of the three exploratory solutions for the Italian MIPS item data.

Given the typical sensitivity of the chi-square tests (χ2) to the sample size, applied SEM research generally focuses on sample size-independent indices to assess model fit [47,48], such as the comparative fit index (CFI), the Tucker–Lewis index (TLI), the root mean square error of approximation (RMSEA), accompanied by a 90% confidence interval (90% CI), and standardized root mean squared residual (SRMR). CFI and TLI values greater than 0.90 and 0.95 are considered a measure of reasonable and excellent model fit, respectively [47]. Likewise, values smaller than 0.08 and 0.06 for RMSEA, and smaller than 0.10 and 0.08 for SRMR, tend to be interpreted as an adequate and good model fit, respectively [47]. Following ESEM analyses, the analyses focused on the internal consistencies of each factor/subscale, as measured by Cronbach’s alpha coefficients (α) and by changes in item–total correlations when an item is deleted (α-item). Cronbach’s alpha values higher than 0.70 are considered ideal [49].

In an additional phase of analyses, the attention was firstly on the temporal stability of the MIPS data collected twice from the subsample of participants (N = 329). This analysis amounted to analyzing the correlations between the two MIPS data over two consecutive months. Secondly, the attention was on construct and criterion validity, for which the statistical analyses focused on the concurrent and/or longitudinal relations between MIPS and both the general measure of perfectionism (F-MPS) and the measure of athletes’ competitive anxiety. With respect to anxiety, which was measured at the latest assessment point, the analyses examined its longitudinal correlations with the MIPS subscales, as well as the possible longitudinal effects yielded by the MIPS subscales. These latter analyses relied on data collected form the subsample of participants (N = 329). These series of analyses were conducted using SPSS, version 26.

## 3. Results

### 3.1. ESEM–Exploratory Structural Equation Modelling

The fit indices of the ESEMs are presented in Table 1. The results showed an unsatisfactory fit (CFI and TLI < 0.90) for ESEM models with seven and eight factors (Models 1 and 2). However, a marginal fit was supported for the nine-factor original model of the MIPS (Model 3). Thus, the standardized loadings of the 72 items of the nine-factor ESEM model were examined.

Upon inspection of the nine-factor solution, it appeared that this solution confirmed seven of the nine factors which characterized the original MIPS factor structure. An eighth factor seemed to combine the two remaining factors of the original MIPS, whereas the ESEM nine-factor solution suggested a ninth “method” factor, which seemed to group items that were semantically related in their wording and that showed secondary loadings. Upon closer inspection, seventeen items (item 6, 7, 14, 15, 20, 21, 28, 29, 38, 39, 46, 47, 54, 55, 58, 64, 69) had statistically significant loadings onto the method factor, with values ranging from −0.08 to a maximum of 0.44 for item 39. These items seem to share the use of adverbs or superlative words (e.g., “extremely”). The method factor seemed thus irrelevant from a theoretical point of view and did not map onto any of the original MIPS factors.

It was then decided to run an additional ESEM analysis including an eight-factor solution and correlated uniqueness for the items loading on the method factor (Model 4). This model provided acceptable goodness-of-fit indices and was chosen as the most ideal solution see Table 1. Item means, standard deviations, skewness and kurtosis, and standardized loadings for the Model 4 are shown in Table 2. The first and second factors included eight items each, all reflecting participants’ perfectionistic aspirations during training (i.e., item 1–8) and during competitions (i.e., item 9–16). The third factor contained sixteen items (i.e., item 17–32), combining the items reflecting participants’ negative reactions to non-perfect performance during training and during competition on a single factor. The fourth, fifth and sixth factors were composed by eight items each, all reflecting participants’ perceived pressure from coach (i.e., item 33–40), parents (i.e., item 49–56) and teammates (i.e., item 41–48), respectively. The seventh factor comprised eight items (i.e., item 57–64), all reflecting participants’ perfectionistic pressure on teammates. Finally, the eighth factor included eight items (i.e., item 65–72), all reflecting participants’ negative reactions to non-perfect performance of teammates. For all eight factors, items loaded significantly and primarily on the expected factor, and all loadings were deemed quite adequate (>0.47).

Descriptive statistics, MIPS scales’ internal consistencies, MIPS inter-scale correlations and MIPS temporal stability coefficients are shown in Table 3 and Table 4. Overall, athletes’ mean scores of perfectionistic aspirations showed, with respect to other MIPS scales, the highest score, and the correlations among all the eight MIPS scales tended to be highly positive and statistically significant (see Table 3). Additionally, all MIPS scales showed excellent Cronbach’s alpha internal consistency coefficients (i.e., all values were above 0.93), and these coefficients did not substantially change when items were deleted to test item-total correlations (see Table 3). Finally, as to the temporal stability of the MIPS scales, each MIPS scale showed positive and significant correlations over the two assessments, as one would expect all coefficients >0.56, see top of Table 4.

### 3.2. Convergent Validity of MIPS

The subsample of 329 participants provided first time assessment data on both MIPS and the F-MPS assessing general perfectionism. It was thus possible to examine the convergent validity of MIPS by assessing patterns of correlations across instruments’ subscales.

As to the patterns of correlations broadly concerned with the MIPS dimension of perfectionistic aspirations, as Table 4 shows, “perfectionistic aspirations during training” and “perfectionistic aspirations during competitions” showed relatively strong positive correlations with “personal standards” and relatively weaker positive correlations with “concerns over mistakes and doubts” and with “organization”. Furthermore, “perfectionistic aspirations during competitions” was the only scale that showed relatively weak correlations with “parental expectations and criticism”.

As to the patterns of correlations broadly concerned with the MIPS dimension of negative reactions to performance, the “negative reactions to non-perfect performance during training and competitions” showed relatively stronger positive relations with “concerns over mistakes and doubts” than with “parental expectations and criticism” and with “personal standards”. There were no statistically significant correlations of the MIPS negative reactions subscale with the F-MPS “organization” subscale.

As to patterns of correlations broadly concerned with the MIPS dimensions of perceived pressure for perfectionism and of personal reactions to non-perfect performance, the scales of “perceived pressure from coach”, “perceived pressure from teammates”, “perfectionistic pressure on teammates” and “negative reactions to non-perfect performance of teammates” showed relatively weak positive correlations with “personal standards”, “concerns over mistakes and doubts” and “parental expectations and criticism”. These MIPS scales did not show any correlation with the “organization” subscale of the F-MPS. Finally, “perceived pressure from parents” showed a relatively strong and positive correlation with “parental expectations and criticism”, whereas it showed a relatively weaker and positive correlation with “personal standards and concern over mistakes”.

### 3.3. The Predictive Validity of MIPS: The Relations with Competitive Anxiety

One of the empirical goals of the study focused on the possible relations of the MIPS dimensions with competitive anxiety. Overall, the expectation was that dimensions of perfectionistic aspirations would show relatively weaker relations with anxiety than would dimensions of personal concerns. The findings supported this general expectation, and this was corroborated by both correlational and regression analyses.

With the first assessment data concerning the concurrent relations between MIPS dimensions with anxiety, the findings in Table 4 showed that “negative reactions to non-perfect performance during training and competitions” had positive correlations with “cognitive” and “somatic” forms of state anxiety and, relatedly, negative correlations with “self-confidence.” Interestingly, the MIPS scales broadly concerned with perceived social pressure for perfection from parents, coaches, and teammates, as well as with concerns for non-perfect personal performance also showed significant, albeit low, positive correlations with cognitive forms of state anxiety. By contrast, the MIPS scales of “perfectionistic aspirations during training”, “perfectionistic aspirations during competitions”, and “perfectionistic pressure on teammates” did not show any significant pattern of correlations with any anxiety dimensions of the CSAI-2.

When these patterns were tested via stepwise regression analyses, the correlational findings were in part confirmed. These analyses tested the possibility that participants’ anxiety assessed at a later point in time would be predicted by MIPS subscales’ scores measured two months earlier (i.e., longitudinal predictions) after controlling statistically for MIPS-anxiety concurrent relations (i.e., first stepwise step). In other words, these stepwise regressions examined longitudinal predictive effects on anxiety yielded by MIPS dimensions.

The stepwise regression findings showed that participants’ early negative reactions to non-perfect performance in training and competition significantly predicted their later cognitive and somatic anxiety, and these effects accounted for a small, yet statistically significant, portion of the total variance in anxiety scores (i.e., R^2^ = 0.029 of 0.215 and R^2^ = 0.022 of 0.158, respectively). As one would have expected, participants’ negative reactions to non-perfect performance also longitudinally predicted lower levels of their self-confidence, and this effect accounted for a small, yet statistically significant, portion of the total variance in confidence scores (i.e., R^2^ = 0.024 of 0.139). The stepwise regression analyses did not yield any longitudinal effect on participants’ anxiety for the MIPS interpersonal dimensions (i.e., the perceived pressure from parents, coaches, and teammates), despite the longitudinal bivariate correlations displayed in Table 4.

## 4. Discussions

The present study primarily focused on the assessment of “perfectionism” in the sport domain and did so by choosing to assess the empirical characteristics of the “personal” and “interpersonal” dimensions that have been originally hypothesized and identified in the scientific validation of the MIPS 72-item instrument [27]. This empirical effort has been carried out among a large sample of Italian sport science students and professional athletes, and its core data analyses relied on the rigorous procedures of exploratory structural equation modeling (ESEM) [38].

Overall, the factor structure of the MIPS item data closely resembled but did not fully replicate the factor structure of the original MIPS item data [27]. In particular, the ESEM analyses confirmed seven of the original nine factors (i.e., two factors of personal strivings, three factors concerning athletes’ perceived pressure from parents, coaches and teammates, and two factors describing athletes’ perfectionism expectations from teammates’ performance and his or her negative reactions to teammates’ non-perfect performance). Additionally, the present study extended prior research with respect to MIPS dimensions concerning teammates, as it not only confirmed their relevance to the underlying factor structure, but also addressed their patterns of convergent relations with other dimensions.

However, there were two relevant findings that were at odd with the original MIPS factor structure. On one side, items referring to the two dimensions of athletes’ “negative reactions to non-perfect performance” during, respectively, training and competition belonged to and loaded onto a single dimension, rather than two separate dimensions, as the original MIPS suggested. On the other, ESEM analyses suggested the presence of a sort of a secondary level “method factor” comprising items that shared semantic and/or wording characteristics. This latter factor was considered irrelevant from a theoretical point of view and was not considered any further in the key analyses.

These findings elicit some considerations. In line with prior research [50], “training” and “competition” stand as relatively distinct contexts in which Italian sport science students and professional athletes may personally strive for, and experience, perfection. On the contrary, training and competition seemingly “blend together” when one considers one’s personal reactions to non-perfect performance or imperfection. That is, while athlete’s perfectionistic strivings may have distinctively different manifestations in “training” and “competitive” settings, his or her personal concerns or negative reactions to non-perfect performance might be common across these two contexts. These considerations notwithstanding, it also is clear that the study findings support the general notion that perfectionistic strivings and perfectionistic concerns represent valid and distinct dimensions of perfectionism as it is experienced personally by athletes.

Thus, perfectionistic strivings and concerns can be viewed as the manifestations of an athlete’s most “personal” and private experience with perfectionism. The present study, by choosing to focus on the validation of the original 72-item MIPS, also assessed the heuristic value of “interpersonal” dimensions of perfectionism, that is, those dimensions in which athletes’ parents, coaches and teammates may act as possible “sources” of pressure towards perfectionistic tendencies.

The ESEM findings of the MIPS items confirmed that coaches, teammates, and parents may represent separate sources of social pressure towards perfectionism also among Italian sport science students and athletes. The present findings are therefore in line with prior studies which were conducted in other geographic or national groups and which only examined social pressure exerted by parents and coaches [29,30]. The findings of the present study also supported the two additional MIPS “interpersonal” dimensions concerning, respectively, participants’ perfectionistic pressure on their teammates and participants’ negative reactions to their teammates’ non-perfect performance. These latter two MIPS dimensions have not explored in other research and the present study thus adds to and extend the original MIPS research [25].

The above considerations on the factor structure or dimensions of the MIPS gained further support from additional findings concerning these dimensions’ measurement characteristics and construct validity. For all MIPS dimensions, item sets had excellent internal consistencies and the dimensions’ scale scores showed quite good stability over the course of two months, thus indicating that systematic individual differences exist in Italian sport science students’ and athletes’ perfectionism.

When the MIPS dimensions were statistically examined with respect to the dimensions of the F-MPS dimensions [40], that is, dimensions that should provide the means for convergent comparisons, the findings of the present study provided further support to the MIPS factor structure. Italian sport science students and athletes who held higher MIPS scores on “perfectionistic aspirations during training” and/or on “perfectionistic aspirations during competitions” also held higher scores on the F-MPS dimension of “personal standards”. Similarly, the study participants who scored higher on “negative reactions to non-perfect performance during training and competitions” also scored higher on the F-MPS scale of “concern over mistakes and doubts” and, even though to a lesser extent, on the scale of “parental expectations and criticism”.

Additional evidence of convergent validity emerged from the patterns of relations of the “interpersonal” MIPS dimensions. In particular, sport science students and athletes who reported relatively high levels of social pressure from their parents, coaches and/or teammates also reported relatively high levels of “parental expectation and criticism”, the only interpersonal dimension of the general perfectionism scale (i.e., F-MPS). Importantly, among these correlational patterns, the highest correlation was between the latter and the MIPS scale of “perceived pressure from parents”. These findings on the convergent validity of the Italian MIPS data are in line with and extend the prior literature in the sport domain [28].

The present study’s choice to validate the original MIPS among Italian athletes was also guided by an interest to assess the possible relations of personal and interpersonal facets of perfectionism with athletes’ competitive state anxiety. The findings of the study yielded patterns supporting the authors’ expectations and the validity of the MIPS in the Italian context. As in previous studies [12,17], athletes’ personal negative reactions to non-perfect performance during either training or competition showed positive relations with cognitive and somatic anxiety and negative relations with self-confidence. These patterns emerged from both correlational and regression analyses. That is, athletes who reported relatively strong concerns about their non-perfect performance also showed relatively strong anxiety and relatively weak self-confidence later in time. Finally, despite the presence of statistically significant longitudinal correlations linking participants’ perceived pressure to perfectionism from significant others and their reported cognitive anxiety, these MIPS interpersonal dimensions did not emerge from more rigorous regression analyses examining the possibility of longitudinal effects on anxiety. These findings only in part confirmed prior research [35]. Taken together, the present study’s findings on anxiety reinforce the general notion that some facets of athletes’ perfectionism might be maladaptive, as other research suggested [17]. More specifically, athletes’ negative reactions or excessive concerns about their non-perfect performance tend to generate thoughts about failure, physical discomfort and nervousness and lack of self-confidence.

### Limitations and Future Research

Despite its substantial value in providing validity of the MIPS among Italian students and athletes, the present study also had several limitations. It did not address possible differences in perfectionism across type and level of sport, even though we collected data on possible differences across athletes on these two variables. The main reason for not performing this type of analysis was concerned with the uneven distribution of the data when the type of sport and level of sport activity were considered simultaneously. In future studies, it will be critical to extend the samples so to minimize the problems of uneven distributions when the type and level of sport need to be examined together. Therefore, any generalization on perfectionism and on its relations with competitive anxiety must be quite cautious. In line with these considerations, recent research [28,31] has stressed the importance of framing future studies of perfectionism within the contexts of specific sports and/or professional agonistic levels.

The present study also implemented a retrospective assessment for measuring athletes’ state anxiety during competitions. Although there is evidence to support athletes’ ability to recall emotions they experienced prior to competition [51], possible failures of memory or subjective reinterpretations of events might have influenced or introduced a method bias in the findings. Future studies might minimize these method problems by closing the time gap between athletes’ recall and their actual competitions.

The present study also primarily relied on correlational data. Its short-term longitudinal component focusing on the assessment of athletes’ competitive anxiety over the course of two months cannot resolve the limits of correlational data, which of course provide no valid means for testing predictive hypotheses about relations between perfectionism and competitive anxiety. Future studies should adopt stronger and more complex longitudinal designs for testing the MIPS validity more rigorously. Finally, future studies should also adopt more thorough assessments of perfectionism by including other instruments that have been developed to address the possibility of domain-specific perfectionism, as other scholars have suggested [52].

## 5. Conclusions

In the sport domain, athletes’ tendency to perfectionism may affect their sport activities, both in training and competitive settings, and this influence might vary significantly depending upon whether perfectionism is experienced at a very personal, private level or, rather, with respect to those who, such as parents, coaches and teammates, may daily influence how athletes experience and react to their sport efforts. The present study attempted to address these notions rigorously and provided the first empirical construct validity evidence of MIPS among Italian sport science students and athletes.

## Figures and Tables

**Table 1:** Goodness of fit indices for the different ESEM models.

	χ2	df	TLI	CFI	RMSEA (90% CI)	SRMR
**Model 1–7 factors**	7227.718	2073	0.801	0.839	0.062 (0.061–0.064)	0.032
**Model 2–8 factors**	6039.272	2008	0.839	0.874	0.056 (0.054–0.057)	0.026
**Model 3–9 factors**	5458.685	1944	0.855	0.890	0.053 (0.051–0.055)	0.022
**Model 4–8 factors-CU**	4716.406	1872	0.878	0.911	0.049 (0.047–0.050)	0.021

Note. N = 644. ESEM: exploratory structural equation modeling; CU: correlated uniqueness; χ2: chi-square test; df: degrees of freedom; TLI: Tucker–Lewis index; CFI: comparative fit index; RMSEA (90% CI): root mean square error of approximation (90% confidence interval); SRMR: standardized root mean square residual.

**Table 2:** Descriptive statistics and factor loadings of the items of the Italian Multidimensional Inventory of Perfectionism in Sport (MIPS).

	Descriptive Statistics				Factor Loadings ESEM–Model 4							
Item	M	SD	Ske	Kur	1	2	3	4	5	6	7	8
1) During training, I feel the need to be perfect.	3.89	1.27			0.70							
2) During training, I strive to be as perfect as possible.	4.52	1.15			0.76							
3) During training, I want to do everything perfectly.	4.52	1.18			0.81							
4) During training, it is important to me to be perfect in everything I attempt.	4.17	1.31			0.77							
5) During training, I demand nothing less than perfection of myself.	3.75	1.42			0.67							
6) During training, I have extremely high expectations of myself. *	3.85	1.34			0.67							
7) During training, I am a perfectionist as far as my targets are concerned. *	3.99	1.36			0.73							
8) During training, I have the wish to do everything perfectly.	4.29	1.33			0.71							
9) During competitions, I feel the need to be perfect.	4.43	1.43				0.73						
10) During competitions, I strive to be as perfect as possible.	4.91	1.27	−1.01			0.82						
11) During competitions, I want to do everything perfectly.	4.79	1.31				0.90						
12) During competitions, it is important to me to be perfect in everything I attempt.	4.52	1.38				0.91						
13) During competitions, I demand nothing less than perfection of myself.	4.10	1.54				0.75						
14) During competitions, I have extremely high expectations of myself. *	4.13	1.42				0.61						
15) During competitions, I am a perfectionist as far as my targets are concerned. *	4.25	1.38				0.70						
16) During competitions, I have the wish to do everything perfectly.	4.64	1.40				0.84						
17) During training, I feel extremely stressed if everything doesn’t go perfectly.	3.05	1.30					0.60					
18) After training, I feel depressed if I have not been perfect.	2.55	1.27					0.66					
19) During training, I get completely furious if I make mistakes.	2.51	1.29					0.54					
20) During training, I set myself such high standards that I cannot fulfill them. *	2.23	1.15	1.08	1.23			0.47					
21) During training, I put myself under pressure with my extremely high expectations. *	2.34	1.25					0.60					
22) After training, I am disappointed if I my performance was not perfect.	2.88	1.24					0.74					
23) If something doesn’t go perfectly during training, I am dissatisfied with the whole training session.	2.61	1.22					0.72					
24) During training, I get frustrated if I do not fulfill my high expectations.	2.61	1.24					0.71					
25) During competitions, I feel extremely stressed if everything doesn’t go perfectly.	2.93	1.36					0.72					
26) After competitions, I feel depressed if I have not been perfect.	2.76	1.31					0.74					
27) During competitions, I get completely furious if I make mistakes.	2.67	1.33					0.63					
28) During competitions, I set myself such high standards that I cannot fulfill them. *	2.28	1.20	1.06				0.54					
29) During competitions, I put myself under pressure with my extremely high expectations. *	2.56	1.33					0.62					
30) After competitions, I am disappointed if I my performance was not perfect.	3.27	1.36					0.67					
31) If something doesn’t go perfectly during competitions, I am dissatisfied with the whole competition.	2.71	1.33					0.75					
32) During competitions, I get frustrated if I do not fulfill my high expectations.	2.74	1.34					0.71					
33) My coach expects my performance to be perfect.	3.30	1.50						0.67				
34) My coach criticizes everything I do not do perfectly.	2.41	1.35						0.76				
35) My coach is dissatisfied with me if my performance is not top class.	2.60	1.36						0.74				
36) My coach expects me to be perfect.	2.98	1.54						0.76				
37) My coach demands nothing less than perfection of me.	2.55	1.50						0.75				
38) My coach makes extremely high demands of me. *	2.33	1.26						0.62				
39) My coach sets extremely high standards for me. *	2.34	1.28						0.63				
40) My coach is disappointed in me if my performance is not perfect.	2.48	1.28						0.67				
41) My teammates expect my performance to be perfect.	2.87	1.49								0.50		
42) My teammates criticize everything I do not do perfectly.	1.98	1.17	1.32	1.38						0.69		
43) My teammates are dissatisfied with me if my performance is not top class.	2.09	1.23	1.12							0.77		
44) My teammates expect me to be perfect.	2.61	1.49								0.63		
45) My teammates demand nothing less than perfection of me.	2.15	1.35	1.08							0.73		
46) My teammates make extremely high demands of me. *	2.01	1.18	1.13							0.76		
47) My teammates set extremely high standards for me. *	2.04	1.27	1.23							0.69		
48) My teammates are disappointed in me if my performance is not perfect.	2.14	1.22	1.03							0.73		
49) My parents expect my performance to be perfect.	1.99	1.30	1.35	1.14					0.67			
50) My parents criticize everything I do not do perfectly.	1.67	1.08	1.90	3.51					0.80			
51) My parents are dissatisfied with me if my performance is not top class.	1.62	1.02	2.03	4.43					0.77			
52) My parents expect me to be perfect.	1.96	1.32	1.41	1.25					0.84			
53) My parents demand nothing less than perfection of me.	1.68	1.14	1.82	2.75					0.87			
54) My parents make extremely high demands of me. *	1.61	1.01	1.95	3.72					0.84			
55) My parents set extremely high standards for me. *	1.63	1.08	1.97	3.55					0.79			
56) My parents are disappointed in me if my performance is not perfect.	1.70	1.06	1.69	2.64					0.78			
57) I demand nothing less than perfection of my teammates.	2.33	1.42									0.71	
58) I have extremely high expectations of my teammates. *	2.78	1.40									0.73	
59) For me, the performance of my teammates has to be perfect.	2.50	1.45									0.89	
60) I expect perfect performance of my teammates.	2.55	1.45									0.90	
61) I want my teammates to do everything as perfectly as possible.	3.22	1.67		−1.09							0.64	
62) It is important to me that my teammates do everything perfectly.	2.80	1.55									0.71	
63) Everything my teammates do has to be of outstanding quality.	2.64	1.44									0.64	
64) I set extremely high standards for my teammates. *	2.24	1.32	1.01								0.53	
65) I am dissatisfied with my teammates, even when I know that they are doing their best.	1.72	1.11	1.77	2.97								0.70
66) If my teammates make mistakes, I consider them failures.	1.52	.97	2.20	5.06								0.74
67) I get annoyed with my teammates if their performance is not first class.	1.74	1.04	1.61	2.62								0.75
68) I get frustrated if my teammates do not fulfill my extremely high expectations.	1.73	1.00	1.43	1.79								0.71
69) I get disappointed if my teammates’ performance is not perfect. *	1.87	1.06	1.35	1.77								0.66
70) I get furious if my teammates’ performance is not top class.	1.69	1.03	1.70	2.81								0.74
71) I cannot stand it when my teammates make mistakes.	1.73	1.01	1.57	2.38								0.70
72) I feel extremely stressed if everything doesn’t go perfectly for my teammates.	1.76	1.04	1.53	2.30								0.62

Note. N = 644. Items with asterisk correspond to CU. Only factor loadings ≥|0.40| are reported. M: mean; SD: standard deviation; Ske: Skewness (values ≤|1| have been suppressed); Kur: Kurtosis (values ≤|1| have been suppressed).

**Table 3:** Descriptive statistics, internal consistency and inter-correlations between the Italian-MIPS subscales.

	M	SD	α-Item	1	2	3	4	5	6	7	8
**1 PA_T**	4.12	1.08	0.92–0.93	(0.94)							
**2 PA_C**	4.47	1.21	0.94–0.95	0.73 **	(0.95)						
**3 NR_TC**	2.67	.96	0.94–0.95	0.36 **	0.38 **	(0.95)					
**4 PPC**	2.63	1.13	0.91–0.92	0.25 **	0.33 **	0.39 **	(0.93)				
**5 PPT**	1.73	.95	0.93–0.94	0.20 **	0.27 **	0.40 **	0.62 **	(0.93)			
**6 PPP**	2.23	1.07	0.92–0.93	0.15 **	0.11 **	0.36 **	0.41 **	0.48 **	(0.94)		
**7 PPOT**	2.63	1.22	0.93–0.94	0.33 **	0.41 **	0.36 **	0.58 **	0.65 **	0.37 **	(0.94)	
**8 NRTT**	1.72	0.85	0.92–0.93	0.08 *	0.09 *	0.43 **	0.40 **	0.57 **	0.53 **	0.57 **	(0.93)

Note. N = 644. * *p* < 0.05; ** *p* < 0.01; M: mean; SD: standard deviation; PA_T: perfectionistic aspirations during training; PA_C: perfectionistic aspirations during competitions; NR_TC: negative reactions to non-perfect performance during training and competitions; PPC: perceived pressure from coach; PPT: perceived pressure from teammates; PPP: perceived pressure from parents; PPOT: perfectionistic pressure on teammates; NRTT: negative reactions to non-perfect performance of teammates; Cronbach’s alpha values are reported in parentheses; α-item: alpha value if an item is removed.

**Table 4:** Test-retest reliability and bivariate correlations among MIPS, MPS and CSAI-2 subscales.

	MIPS							
	PA_T	PA_C	NR_TC	PPC	PPT	PPP	PPOT	NRTT
M (SD)	4.09 (1.05)	4.42 (1.18)	2.71 (0.93)	2.66 (1.14)	2.26 (1.09)	1.78 (0.97)	2.62 (1.19)	1.76 (0.87)
r_t–r_	0.64 **	0.65 **	0.61 **	0.64 **	0.56 **	0.62 **	0.60 **	0.57 **
**MPS**								
PS	0.50 **	0.43 **	0.15 **	0.24 **	0.15 **	0.23 **	0.28 **	0.12 *
CMD	0.16 **	0.19 **	0.41 **	0.14 **	0.15 **	0.21 **	0.14 **	0.11 *
PEPC	0.08	0.13 *	0.36 **	0.26 **	0.27 **	0.51 **	0.26 **	0.30 **
O	0.22 **	0.13 *	0.02	0.03	0.01	0.05	0.07	−0.07
**CSAI-2**								
CA	0.06	0.08	0.40 **	0.12 *	0.17 **	0.13 *	0.07	0.11 *
SA	0.04	0.04	0.32 **	0.07	0.07	0.09	0.03	0.03
SC	0.10	0.07	−0.21 **	0.07	0.04	0.02	0.09	0.05

Note. N = 329. * *p* < 0.05; ** *p* < 0.01; M (SD): mean (standard deviation); r_t–r_: test-retest correlation after nearly two months; MPS: multidimensional perfectionism scale; PS: personal standards; CMD: concern over mistakes and doubts; PEPC: parental expectations and criticism; O: organization; CSAI-2: competitive state anxiety inventory-2; CA: cognitive anxiety-state; SA: somatic anxiety-state; SC: self-confidence; PA_T: perfectionistic aspirations during training; PA_C: perfectionistic aspirations during competitions; NR_TC: negative reactions to non-perfect performance during training and competitions; PPC: perceived pressure from coach; PPT: perceived pressure from teammates; PPP: perceived pressure from parents; PPOT: perfectionistic pressure on teammates; NRTT: negative reactions to non-perfect performance of teammates.

## Data Availability

The data presented in this study are available on request from the corresponding author.
